# Orofacial symptoms and oral health-related quality of life in juvenile idiopathic arthritis: a two-year prospective observational study

**DOI:** 10.1186/s12969-018-0259-4

**Published:** 2018-07-13

**Authors:** Hanna Rahimi, Marinka Twilt, Troels Herlin, Lynn Spiegel, Thomas Klit Pedersen, Annelise Küseler, Peter Stoustrup

**Affiliations:** 1 Department of Pediatrics, Aarhus University Hospital, Palle Juul-Jensens Boulevard 121, 8200 Aarhus N, Denmark; 2 Department of Pediatrics, Division of Rheumatology, Alberta Children’s Hospital, 2888 Shaganappi Trail NW, Calgary, AB Canada; 3 Division of Rheumatology, The Hospital for Sick Children, 555 University Avenue, Toronto, ON Canada; 4 Department of Oral and Maxillofacial Surgery, Aarhus University Hospital, Nørrebrogade 44, 8000 Aarhus C, Denmark; 5 Section of Orthodontics, Department of Dentistry and Oral Health, Aarhus University, Vennelyst Boulevard 9-11, 8000 Aarhus C, Denmark

**Keywords:** Juvenile idiopathic arthritis, Temporomandibular joint, Quality of life, Pain, Oral health, Orofacial symptoms, Health assessement questionaire

## Abstract

**Background:**

Little is known about the chronicity of orofacial symptoms and how this influences the oral health-related quality of life in juvenile idiopathic arthritis (JIA). Therefore, our objectives were to study the long-term changes in self-reported orofacial symptoms, and to define the impact of orofacial symptoms on oral health-related quality of life in JIA.

**Methods:**

At baseline (T0), 157 consecutive JIA patients ≤20 years completed a patient pain questionnaire that incorporates domains related to the orofacial area. At the 2 year follow-up (T1), 113 patients completed the same questionnaire (response rate 72%) in addition to the Child Perception’s Questionnaire; a validated 31-item questionnaire addressing oral health-related quality of life.

**Results:**

At T0, 53% (60/113) of patients reported the presence of orofacial pain, and 36% (41/113) of patients reported compromised orofacial function. At T1, 77% (46/60) of patients with pain at T0 reported persistent pain, and 66% (27/41) of patients with functional disability at T0 reported persistent disability. Patients with orofacial symptoms reported a significantly greater prevalence of negative impact of orofacial conditions on general quality of life and within the domains of emotional and social well-being compared to asymptomatic patients.

**Conclusion:**

Self-reported orofacial pain and functional disability were common findings in a cohort of JIA patients followed over 2 years. These symptoms seem to persist over time in most patients, and have a significant negative impact on oral health-related quality of life.

**Electronic supplementary material:**

The online version of this article (10.1186/s12969-018-0259-4) contains supplementary material, which is available to authorized users.

## Background

Involvement of the temporomandibular joint (TMJ) is a common finding in patients with juvenile idiopathic arthritis (JIA) [1, 2]. Long-term arthritis of the TMJ may lead to growth dependent deformation of the joint components and reduced joint mobility, which in turn, may lead to secondary compromise of TMJ function and related muscular structures [3–7]. Arthritis-induced orofacial signs and symptoms are common entities in JIA, and are associated with young age at onset, long disease duration, involvement of upper extremities, and polyarticular and systemic JIA subtypes [3, 8, 9]. Across the literature, the reported prevalence of orofacial symptoms in JIA varies greatly, possibly due to the differences in the included cohort characteristics, retrospective character of most studies, and the type of questions asked [7].

Generally, there is a lack of knowledge of the long-term chronicity of orofacial symptoms in JIA. Although follow-up studies exist, the current knowledge of JIA-induced orofacial symptoms mainly originates from cross-sectional studies [4]. Long-term observational studies by Bakke et al. (15 year follow-up) and Engstrom et al. (25 year follow-up) have outlined a high prevalence of patients with persistent JIA-induced orofacial signs and symptoms [10, 11]. However, these studies represent JIA cohorts from the pre-biologic era, which are incomparable to the contemporary JIA cohorts receiving targeted therapy [12]. A small prevalence of patients with persistent orofacial symptoms were described in a 5-year follow-up study by Twilt et al. in 2008 [13]. In support of that, a longitudinal study by Zwir et al. from 2015 found a baseline prevalence of orofacial symptoms of 29%, with a reduction to 12% at 1 year follow-up [14]. Therefore, only limited knowledge is available on the long-term nature of orofacial symptoms in contemporary JIA patients. Additionally, little knowledge is available of the impact of JIA-induced orofacial symptoms on quality of life specifically related to the orofacial area. Previous cross-sectional studies by Leksell et al. and Frid et al. have focused on the association between arthritis-induced orofacial symptoms and general health-related quality of life using the Childhood Health Assessment Questionnaire (CHAQ) and the Child Health Questionnaires (CHQ) [9, 15, 16]. These questionnaires assess general impact of arthritis and are not tools specifically designed to assess the impact of orofacial dysfunction on parameters related to oral health.

The purpose of this prospective observational cohort-study was: 1) To study the long-term changes in self-reported orofacial symptoms, 2) To study the impact of orofacial symptoms on oral health-related quality of life (OHRQOL). We hypothesized that the presence of orofacial symptoms would have a significant impact on the OHRQOL.

## Methods

This prospective observational study was conducted at the Section of Orthodontics, Department of Dentistry and Oral Health, Aarhus University, Denmark in the period between 2014 and 2017. Patients with JIA are referred to the Aarhus University orthodontic clinic from all pediatric rheumatology hospital centers in Western Denmark and are followed longitudinally regardless of presence or absence of TMJ arthritis. Therefore, the entire cohort seen at the Section of Orthodontics is a representation of the JIA population of Denmark. In 2014 and 2015, consecutive patients were invited to participate in the study. Participants completed a self-report questionnaire that assesses orofacial symptoms in JIA. In 2017, all patients from the baseline study were invited to participate in a two-year follow-up survey. Inclusion criteria were: 1) JIA-diagnosis according to the criteria of the International League of Associations for Rheumatology (ILAR) [17], 2) cognitively capable of understanding and answering the questionnaires, 3) ≤20 years old when completing the baseline questionnaire.

### The patient questionnaires

At baseline (T0), consecutive patients were asked to complete a standardized patient questionnaire concerning symptoms within the last 2 weeks. The questionnaire was created in accordance with international consensus-based recommendations for orofacial assessment in JIA [7], and incorporates the following domains: 1) orofacial pain frequency assessed by a 5 point Likert scale (0 = “never”, 1 = “less than once a week”, 2 = “several times a week”, 3 = “several times a day”, 4 = “all the time”); 2) orofacial pain intensity, for which a 100 mm VAS was used (0 = “not affected”, 100 = “severely affected”); 3) orofacial pain location, assessed by letting the participant mark the area of pain on a diagram illustrating the head and neck; 4) orofacial functional disability (100 mm VAS, 0 = not affected, 100 = severely affected); and 5) characteristics of orofacial symptoms, assessed by asking participants to mark off all the statements that were applicable to them. To combine aspects of orofacial pain intensity and frequency into one single outcome measure, we calculated a composite pain index variable by multiplying pain frequency and pain intensity with a score range between 0 and 400. In addition, with reference to the pain intensity and frequency for the 2 weeks subsequent to the study visit, we asked the participants to assess their global pain score based on a 100 mm VAS (Endpoints: 0 = no pain, 100 = worst imaginable pain).

At follow-up (T1), the patients were asked to complete the same questionnaire that they had completed at T0. In addition, they were asked to complete a validated 31-item questionnaire addressing OHRQOL (Child Perception’s Questionnaire) [18]. The questionnaire includes two global ratings: 1) Self-reported perception of own oral health status, 2) The extent to which the orofacial conditions affect the overall general quality of life. In addition, the questionnaire contains 29 questions related to general emotional and social well-being (see online Additional file 1). Information about medical treatment and TMJ arthritis-related treatment between T0 and T1 was collected from chart files. The study was approved by the Danish Data Protection Agency (2016–051-000001, ID:665) and conducted in agreement with Danish Health authority regulations on questionnaire-based studies and chart-files studies; Informed and signed consent was provided by all participants ≥15 years of age, and by their parents for participants below age 15.

### Statistics

Graphical display revealed that numerical variables (pain intensity, pain index and functional disability) were skewed and not normal distributed. All numerical and categorical variables were analyzed using Wilcoxon’s rank-sum tests for paired data variables and Mann-Whitney tests for unpaired data. Chi-square tests were used to assess changes in the prevalence of the specific characteristics of orofacial symptoms between T0 and T1. Agreement between the pain index composite variable (pain frequency x pain intensity) and patient global pain score was calculated using the Bland-Altman analysis [19]. Correlation coefficients were used to assess the correlation between the pain index variable global ratings of self-reported perception of own oral health status and the impact of orofacial conditions on general quality of life. The level of significance was 0.05. Statistical analysis was conducted using the Stata 13 software (StataCorp. 2013. Stata Statistical Software: Release 13. College Station, TX: StataCorp LP)

## Results

At T0, 157 eligible consecutive patients completed the questionnaire. All 157 patients were invited to participate in the two-year follow-up questionnaire survey. At T1 (mean 25 months, SD 3.1 months), 113 patients accepted the invitation and repeated the questionnaire (response rate 72%). Only patients who completed the questionnaires at both time points were included in the present study. The characteristics of the 113 study patients are presented in Table 1. The most frequent JIA subcategories with baseline orofacial symptoms were oligo persistent JIA (48%) and polyarticular JIA (39%). In addition, Table 1 displays the treatments conducted between T0 and T1. No significant differences in mean age or disease duration at T0 were seen between patients with or without orofacial symptoms. A baseline analysis showed no inter-group differences in the reported symptoms between included patients who completed the questionnaires at both time-points and excluded patients who only completed the baseline questionnaire.

**Table 1:** Patient Characteristics

Cohort characteristics	Orofacial symptomatic group at T0	Orofacial asymptomatic group at T0
Number	62	51
Mean age at baseline, years (sd)	14.6 (2.9)	13.9 (2.4)
JIA subcategories, number		
Oligoarticular extended	4	1
Oligoarticular persistent	26	28
Polyarticular	27	17
Systemic	1	1
Psoriatic	3	4
Enthesitis related arthritis	0	0
Unknown	1	0
Disease duration		
Mean years (sd)	7.4 (4.2)	6.6 (4.4)
< 1 years	0	1
0–3	5	6
> 3	57	44
TMJ-specific treatment in the follow-up period, number		
Flat splint (night)	12	5
Flat splint (full time)	10	0
Distraction splint	4	6
Full fixed appliances	5	4
Previous full fixed appliances	12	9
Surgical osseous distraction	1	0
Activator	4	3
Intra articular TMJ steroid	3	0
Orofacial physiotherapy	8	0
Home Exercises	4	0
No treatment	12	25
Medical treatment in the follow-up period, number		
NSAID	19	4
Methotrexate	27	16
Leflunomide	4	0
Systemic steroid	1	0
Biologics	26	16
No medication	36	38
Combination of two drugs	19	6
Combination of three drugs	3	1
Change in treatment during follow-up	21	15

Characteristics of study patients. TMJ: Temporomandibular joint

### General findings

**Fig. 1:**
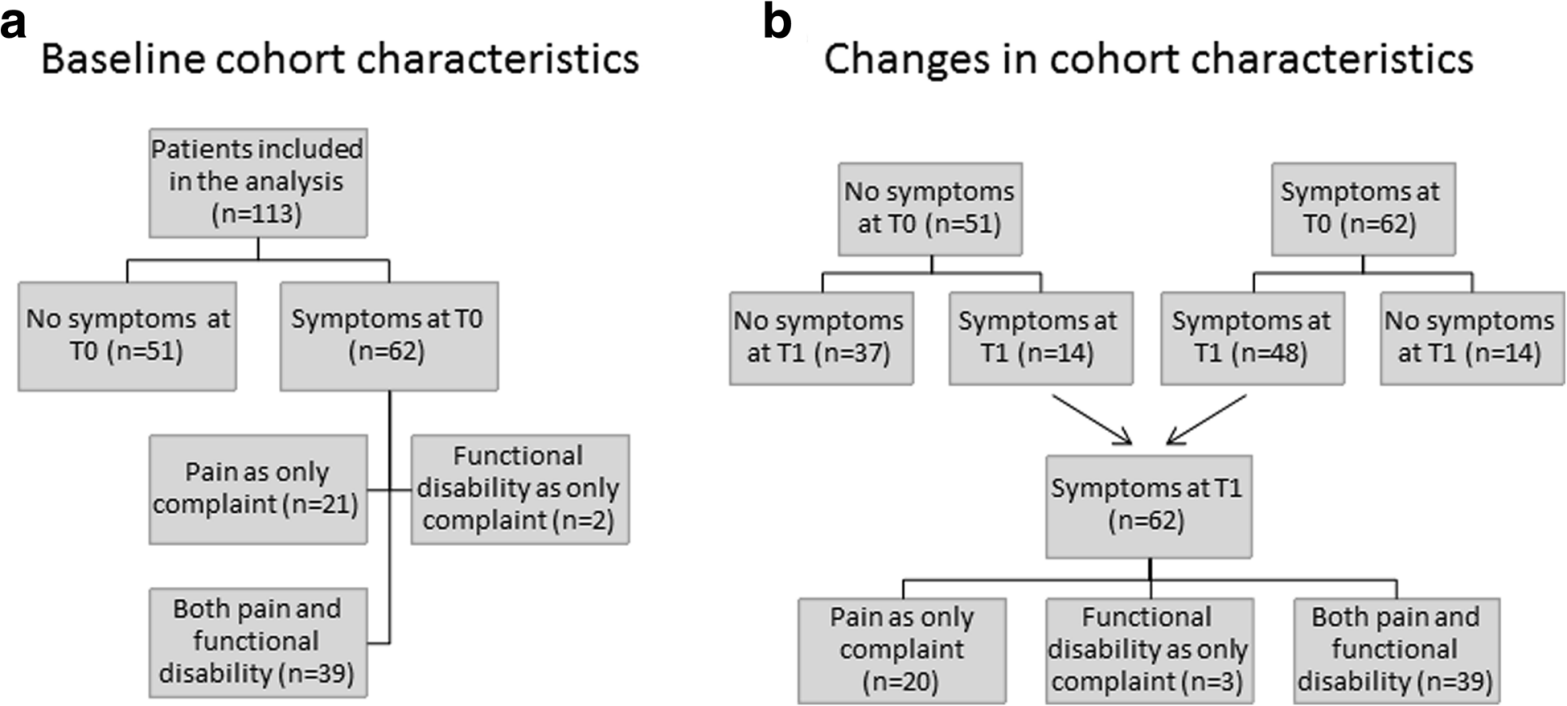
**a** Description of cohort baseline (T0) orofacial symptoms. **b** Changes in orofacial symptoms between baseline (T0) and the two-year follow-up (T1)

At T0, 55% of patients (62/113) reported the presence of orofacial symptoms and 45% of patients (51/113) were asymptomatic (Fig. 1a). The majority of symptomatic patients experienced both pain and functional disability (63%, 39/62). A smaller number of symptomatic patients experienced pain only (34%, 21/62) or functional disability only (3%, 2/62) (Fig. 1a).

At T1, 77% (48/62) of patients reported “persistent symptoms” indicated by a report of symptoms at both time points (T0 and T1). Between T0 and T1, 27% (14/51) of patients developed new symptoms; 7% (1/14) reported functional disability only; 50% (7/14) reported pain only; and 43% (6/14) reported both pain and functional disability. Twenty-three percent (14/62) of patients with symptoms at T0 experienced a resolution of symptoms at T1 (Fig. 1b).

### Pain frequency

**Fig. 2:**
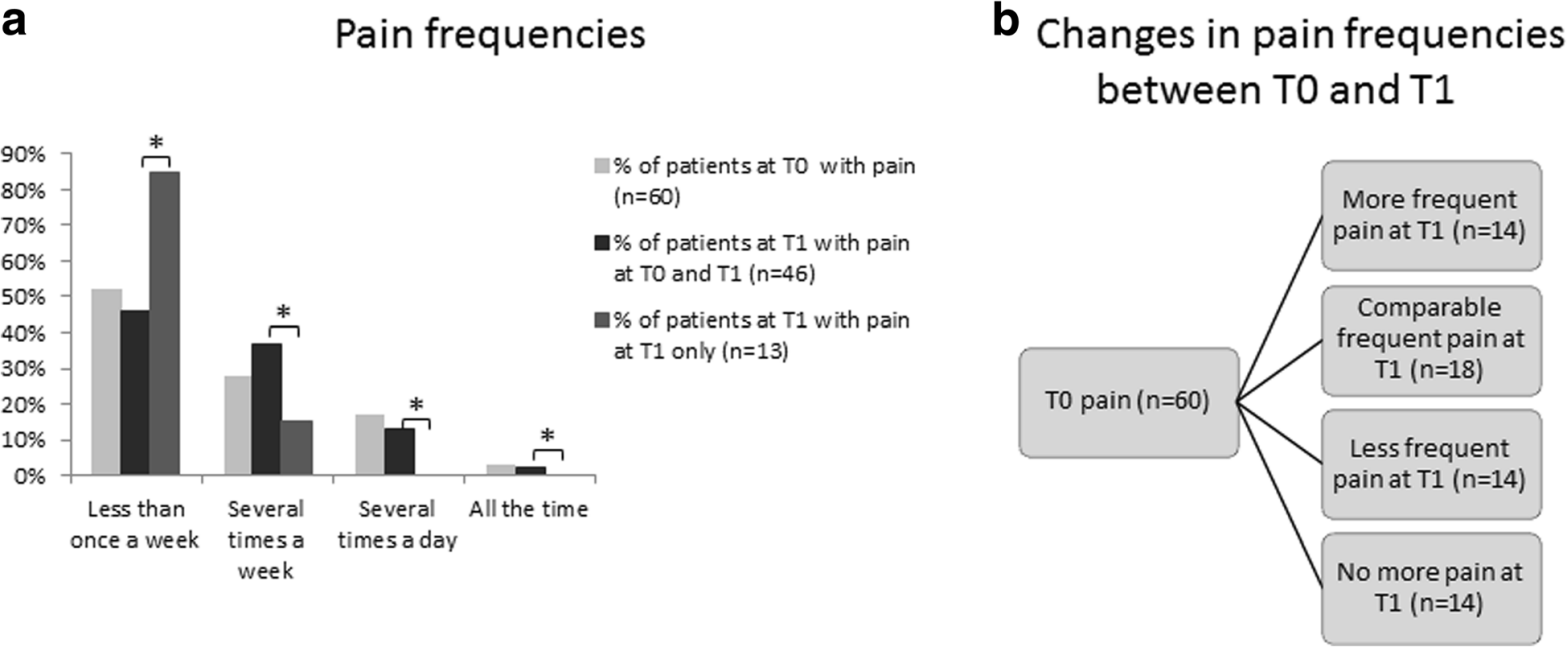
**a** Pain frequencies in patients with pain at baseline (*n* = 60), pain at T0 + T1 at follow-up (*n* = 46) and, pain at follow-up only (*n* = 13) * = Subjects with persistent pain (T0 + T1) reported significantly higher frequencies of pain than patients with pain at T1 only, **b** Changes in pain frequencies between T0 and T1 in patients with pain at baseline (n = 60)

Orofacial pain frequencies for the different time-points are displayed in Fig. 2a. At T0, 53% (60/113) of patients reported the presence of orofacial pain. Almost half of patients with orofacial pain (29/60) reported pain on a weekly basis. Seventy-seven percent (46/60) of patients with pain at T0 also reported pain at T1 (Fig. 1b). Changes in pain frequency observed between T0 and T1 were: 30% (14/46) reported less frequent pain at T1; 39% (18/46) reported comparable pain frequency at T0 and T1; and 30% (14/46) reported more frequent pain at T1 (Fig. 2b). The remaining 23% (14/60) of patients with pain at T0 reported no pain at T1. There was no significant difference in the reported T0 pain frequencies between the 14 patients who only experienced pain at T0, when compared to the 46 patients with persistent pain complaints. At T1, patients with persistent pain (*n* = 46) reported significantly higher pain frequencies than patients with newly developed pain between T0 and T1 (*n* = 13) (Fig. 2a).

### Pain intensity

**Fig. 3:**
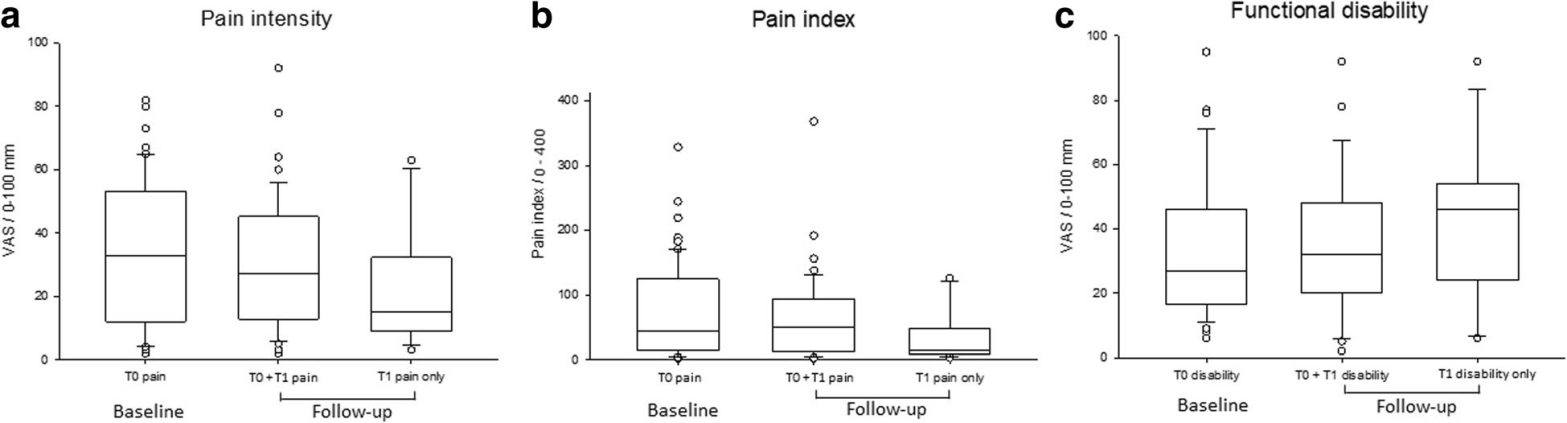
**a** VAS-scores of pain intensity (VAS 0–100 mm). **b** Pain index (pain frequency x pain intensity, range 0–400). **c** Functional disability (VAS 0–100 mm), in patients with pain at baseline (n = 60), at T0 and T1 at follow-up (n = 46) and at follow-up only (n = 13). In 3abc, baseline represents the total group of patients with reports of symptoms. Follow-up represents two groups: 1) Patients with pain (Fig. 3ab) or functional disability (3c) at both T0 and T1), 2) patients with reports of pain (Fig. 3ab) or functional disability (3c) at follow-up only

A change in pain intensity between T0 and T1 was defined as a VAS scale difference ≥ 13 mm in accordance with the smallest detectable difference for average orofacial pain reports, as previously described [6]. At T0, the median and the inter-quartile ranges (IQR) between the 1st and 3rd quartiles for orofacial pain intensity was 33 mm (IQR = 12–52.5 mm, *n* = 60) (Fig. 3a). A non-significant difference in pain-intensity was observed between T0 and T1 in patients with reports of persistent pain. The change in pain intensity between T0 and T1 for patients with persistent pain was as follows: 37% (17/46) reported less intense pain at T1, 39% (18/46) reported comparable pain intensity at T0 and T1, and 24% (11/46) reported more intense pain at T1. Fourteen patients with pain at T0 reported no pain at T1. There was no significant difference in the reported pain intensities at T0, between the 14 patients with pain at T0 only, when compared to the 46 patients with pain complaints at both T0 and T1.

At T1, a non-significant higher median pain intensity of 27 mm (IQR = 13-45 mm, *n* = 46) was reported by patients with persistent pain when compared to the median pain intensity of 16 mm (IQR = 12–38 mm, *n* = 13) reported by patients with pain at T1 only (Fig. 3a).

### Pain index

The correlation coefficient between the pain index composite variable (pain frequency x pain intensity) and patient global pain score was *r* = 0.78 indicating an acceptable validity of the pain index variable as a measure of the general pain perception of the patients.

At T0, the median orofacial patient pain index (*n* = 60) was 43.5 (IQR = 15–121, n = 60) (Fig. 3b). At T1, a non-significant higher median pain index of 50 (IQR = 13–94) was reported by patients with persistent pain (*n* = 46) when compared to a median 16 (IQR = 12–45) pain index reported by patients with pain at T1 only (*n* = 13). No significant changes in pain index values were observed between T0 and T1 in patients with persistent pain reports (Fig. 3b).

### Pain location

**Fig. 4:**
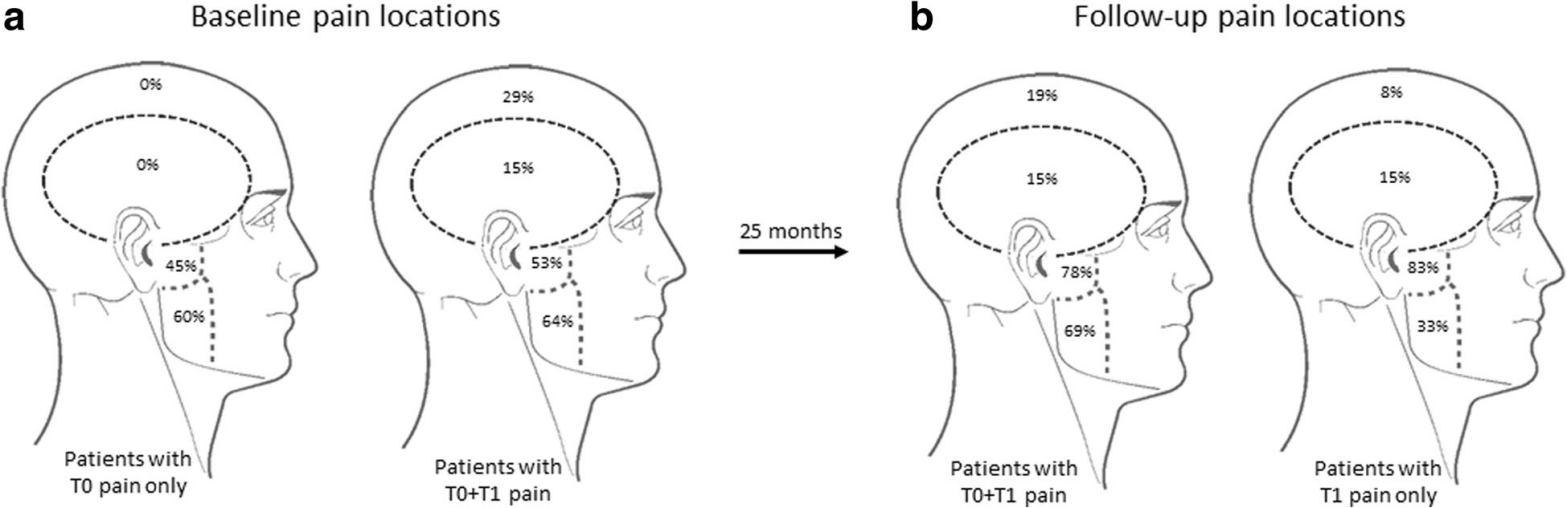
**a** Distribution of orofacial pain at baseline among patients with T0 pain only and patients with pain at T0 and T1. **b** Distribution of orofacial pain at follow-up among patients with pain at T0 and T1 and patients with T1 pain only

Figure 4a illustrates the distribution of the orofacial pain at T0. Patients with pain at T0 only reported pain in TMJ and masseter muscle regions exclusively. In contrast, patients with persistent pain reported a more widespread pain distribution at T0, involving the temporal, frontal and parietal regions. Multiregional pain was reported by 46% (21/46) of patients with persistent orofacial pain and by 7% (1/14) of patients with orofacial pain at T0 only. There was no change in the distribution of pain locations seen between T0 and T1 in patients reporting persistent pain (Fig. 4b). Patients with persistent pain (n = 46) reported a significantly higher prevalence of masticatory muscle pain when compared to patients who reported pain at T1 only (n = 13): All other pain locations were involved to a comparable degree in these two groups (Fig. 4b). Generally, the most involved pain locations at both T0 and T1 were the TMJ and the masseter muscle regions.

### Functional disability

At T0, the median level of VAS-reported functional disability was 27 mm (IQR = 17-46 mm, *n* = 41) (Fig. 3c). Ninety-five percent (39/41) of patients reporting T0 functional disability also reported orofacial pain (Fig. 1a). At T1, a non-significant median level of functional disability of 32 mm (IQR = 20-48 mm) was reported by patients with persistent functional complaints (*n* = 27/41) when compared to a median of 46 mm (IQR = 24-54 mm, *n* = 15) in patients reporting functional disability at T1 only (Fig. 3c). The non-significant changes in functional disability scores between T0 and T1 were as follows: 26% (7/27) reported an improvement of orofacial functional disability between T0 and T1, 48% (13/27) reported the same level of orofacial functional disability, and 26% (7/27) reported a worsening of orofacial functional disability at T1. Ninety-three percent (25/27) of patients reporting persistent orofacial function disability also reported orofacial pain at both time points.

### Symptoms

**Fig. 5:**
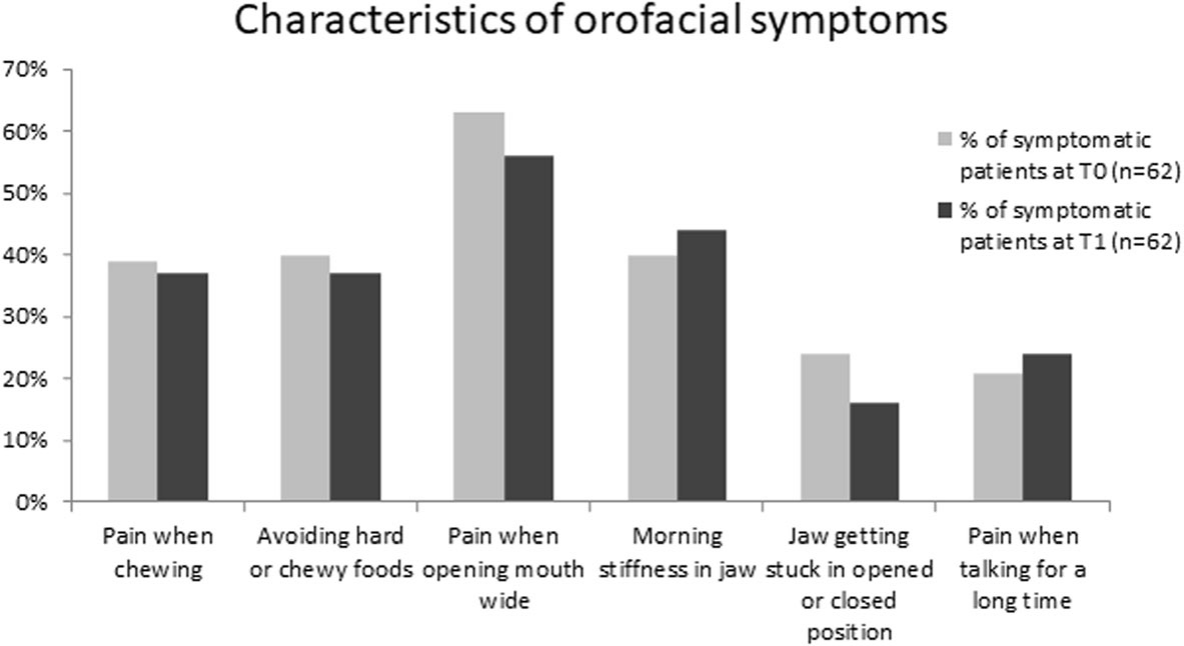
Characteristics of orofacial symptoms in patients with orofacial pain and/or functional disability at T0 (*n* = 62) and T1 (n = 62). No significant difference in characteristics of orofacial symptoms between baseline and follow-up

The characteristics of orofacial symptoms reported at T0 and T1 are displayed in Fig. 5. The majority of patients reported pain when opening the mouth wide (63% at T0 and 56% at T1). Other frequent complaints were jaw morning stiffness (40% at T0 and 44% at T1), pain when chewing (39% at T0 and 37% at T1) as well as avoiding hard or chewy foods (40% at T0 and 37% at T1). No significant differences were found in the distribution of symptoms between T0 and T1.

### OHRQOL

**Fig. 6:**
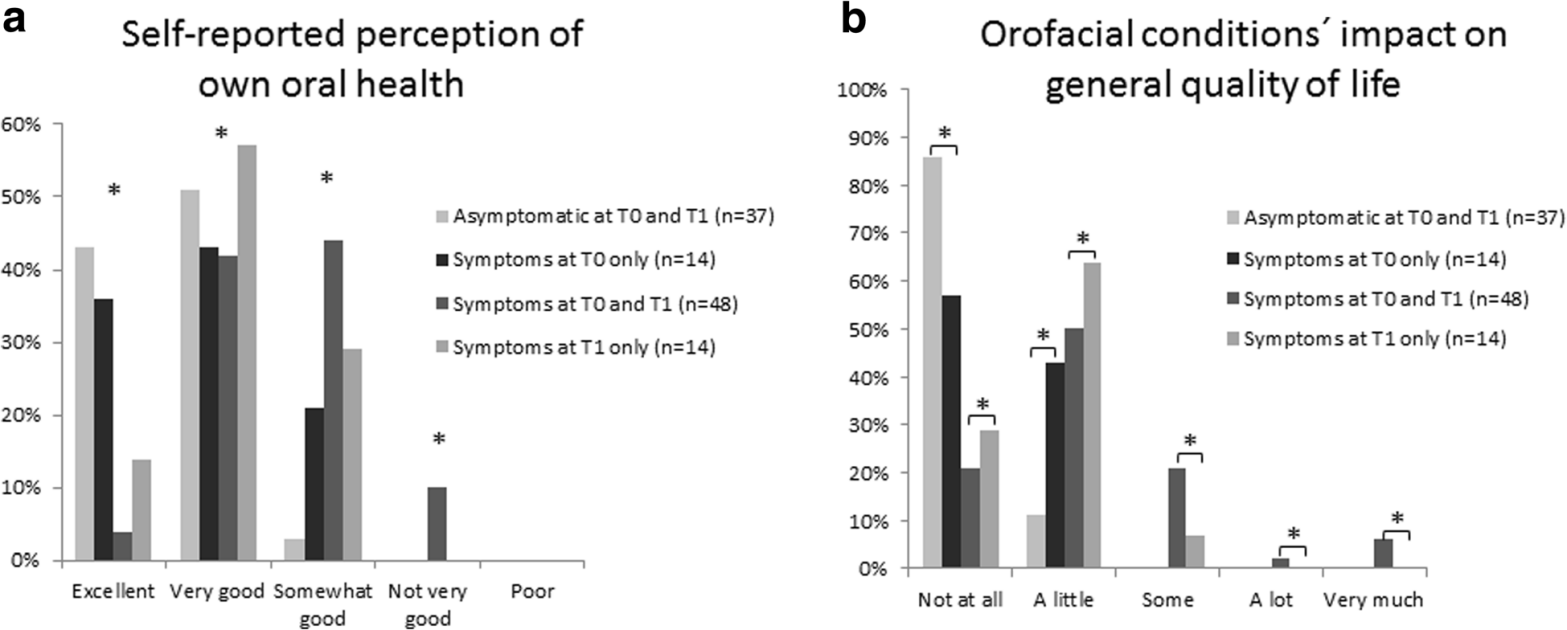
**a** Self-reported perception of own oral health. * = significantly reduced perception of own health in patients reporting symptoms as compared to asymptomatic patients. **b** orofacial conditions’ impact on general quality of life, in asymptomatic patients at T0 and T1 (*n* = 37), symptomatic patients at T0 only (*n* = 14), symptomatic patients at T0 and T1 (*n* = 48) and symptomatic patients at T1 only (n = 14). * = significant intergroup differences

The global rating of self-reported perception of own oral health was significantly reduced in symptomatic as compared to asymptomatic patients at T1 (Fig. 6a). A subgroup analysis revealed that no significant differences in self-reported perception of oral health were found between patients with persistent symptoms (T0 and T1), when compared to patients who only reported symptoms at T1. Asymptomatic patients and patients with symptoms at T0 only reported comparable perceptions of oral health (Fig. 6a). A low correlation of *r* = 0.32 was found between the pain index variable and global rating of self-reported perception of own oral health in patients with symptoms at T1, indicating a limited association between the severity of orofacial pain and the self-reported rating of own orofacial health.

The impact of orofacial conditions on general quality of life was significantly higher in patients reporting symptoms as compared to asymptomatic patients at T1 (Fig. 6b). A subgroup analysis revealed a significant difference in the impact of orofacial conditions on the general quality of life in patients with persistent symptoms when compared to patients who only reported symptoms at T1. Patients with orofacial symptoms at T0 and no symptoms at T1 reported a significantly higher impact of orofacial conditions on their quality of life compared to patients who were asymptomatic at both time points. Eighteen percent (11/62) of patients with orofacial pain and/or functional disability at T1 reported that the condition had “some” negative impact on the overall quality of life. Six percent (4/62) of symptomatic patients at T1 reported that the orofacial condition reduced their general quality of life “a lot” (1/62) and “very much” (3/62). A moderate correlation of *r* = 0.54 was found between pain index values and the self-reported impact of orofacial condition on general quality of life in patients with symptoms at T1.

### Emotional and social well-being

The impact of items related to emotional and social well-being among patients with and without TMJ-arthritis symptoms are presented in the online Additional file 1. Patients with orofacial symptoms reported a significantly greater prevalence of negative impact on questions related to emotional and social well-being.

## Discussion

To our knowledge, this is the most comprehensive longitudinal study examining orofacial symptoms in JIA. The objective of this study was to study the long-term changes in self-reported orofacial symptoms and to study the impact of orofacial symptoms on OHRQOL. The findings of this study demonstrate: Orofacial symptoms are common findings in patients with JIA, and they tend to persist with time. Furthermore, the intensity, frequency and the characteristics of orofacial symptoms do not change significantly over time. The TMJ and masseter regions are the most frequent orofacial areas affected, however, multiregional orofacial pain was seen in a substantial number of patients with persistent symptoms. Orofacial pain is associated with functional disability in the majority of patients, and it is rare to see functional disability in the absence of orofacial pain. We found that the pain index composite variable (pain frequency x pain intensity) is an acceptable measure of patient global pain perception. Patients with orofacial symptoms reported a significantly higher negative impact of orofacial conditions on general quality of life compared to asymptomatic patients. Finally, patients with orofacial symptoms reported a significant negative impact on emotional and social well-being.

This study found a high prevalence (55%) of JIA patients with orofacial pain and dysfunction. This is in contrast to a previous 5-year follow-up study by Twilt et al., who reported a smaller prevalence of orofacial pain (13%) and limited mandibular function (10%) [13]. In our study, 97% (60/62) of symptomatic patients at T0 experienced orofacial pain and 77% (46/60) of these patients still reported pain after 2 years (T1). This finding is in contrast with Engstrom et al. who reported a higher prevalence of orofacial symptoms over time in their 15 year follow-up study [11]. In agreement with Frid et al. we found an increased prevalence of orofacial symptoms in patients with a polyarticular disease course [9].

The nature of the questionnaire used in this study allowed for a comprehensive analysis of changes in orofacial pain characteristics in JIA over time. In the group experiencing pain at both T0 and T1, we did not observe a specific pattern for changes in pain frequency or pain intensity over time. The current literature lacks information about orofacial pain frequency in JIA [4]. This study therefore contributes valuable information by demonstrating that daily/weekly pain fluctuation is a characteristic finding in JIA patients who report orofacial symptoms. Many patients experienced orofacial symptoms during mastication and maximal mouth opening maneuvers. However, when asked about pain frequency, full-time symptoms were rarely reported, and the majority did not experience orofacial symptoms every day. From a clinical point of view, this is important because it conflicts with existing standardized guidelines on clinical orofacial assessment like the DC/TMD criteria, which was not exclusively developed for JIA [20]. A critical tenet of the DC/TMD criteria is the notion that arthralgia, can only be established if pain on palpation is present during the clinical examination [20]. Applied to a JIA population, this would mean an under reporting of orofacial pain, since many patients only experience pain in conjunction with functional demands like mastication. To capture the fluctuation of orofacial pain in JIA, we introduced the pain index variable (pain frequency x pain intensity). The acceptable agreement between the pain index variable and the patient global pain score of *r* = 0.78 reveals that this may be a useful variable to address the fluctuation of orofacial pain in JIA. Interestingly, the reported T0 and T1 median pain index scores were surprisingly small considering the range 0–400 of the pain index outcome measure.

In the present study, patients reporting persistent T1 pain reported more widespread pain distribution as well as higher prevalence of multiregional pain compared to those reporting pain at T0 only. Notably, the locations of the affected pain regions and the characteristics of orofacial symptoms did not significantly change over time in patients with persistent orofacial symptoms.

In this study, the presence of orofacial pain and/or functional disability significantly impacted general health related quality of life. This is in agreement with a previous study by Leksell et al. and Frid et al. [9, 15] but contrasts with findings of Twilt et al. [13] who reported no significant impact on general quality of life between patients with and without TMJ involvement. However, an only moderate association (*r* = 0.54) between the pain index variable and the impact of an orofacial condition on general quality of life demonstrates that a high level of orofacial pain may not negatively impact general quality of life and vice versa.

Patients with persistent orofacial symptoms experienced a greater impact on their general quality of life compared to patients with symptoms at T1 only. Although we do not have any information about pain related symptoms between those two observation points, this suggests that long-term symptoms impact general quality of life to a greater degree than short-term orofacial symptoms. Moreover, we also observed that patients who only had symptoms at T0 reported a significantly greater impact of orofacial conditions on general quality of life at T1 when compared to asymptomatic patients. This is an interesting finding, and may indicate that previous orofacial symptoms can impact general quality of life even after symptoms have resolved. However, the present study does not allow us to make firm conclusions in this regard. Future work with a larger patient cohort studied at more frequent time intervals could help to clarify some of these findings.

In the present study, we used a validated questionnaire to assess domains related to emotional and social well-being [18]. This is the first study to assess oral health related quality of life in JIA. We found that emotional and social well-being were significantly reduced in patients with orofacial symptoms. Currently no validated OHRQOL questionnaire exists exclusively for use in JIA which constitutes a limitation to the present study. The questionnaire used, in the present study, has been validated in non-JIA children and adolescence with “orofacial conditions” (18). This warrants a future validation of OHRQOL questionnaires exclusively to the JIA population.

Our cohort consisted of consecutively enrolled JIA patients, from the entire JIA population in Denmark, thus decreasing the risk for selection bias at T0. Although the background cohort reflects the JIA population in Denmark, it should be noted that there were no patients with enthesitis-related arthritis and undifferentiated arthritis completing the questionnaires at both time-points in the present study. Furthermore, standardized questionnaires were used assessing orofacial symptoms and OHRQOL, thus minimizing the risk of information bias. There were however some limitations to this study. The current data does not contain information about presence/absence of TMJ inflammation at the time the questionnaires were completed; this would have been important information to collect. However, this is likely of minor significance since previous studies have revealed that the presence of orofacial pain is a weak predictor of TMJ arthritis [4]. When interpreting symptoms, we typically attributed orofacial symptoms to previous TMJ arthritis leading to structural damage and impaired TMJ function. However, orofacial symptoms are also seen in non-inflammatory temporomandibular disorders, a common finding in the general population, and thus a potential confounder to the prevalence of symptoms reported in this study [21]. Therefore, a general limitation to the present study is the lack of a non-JIA control group to reflect the frequency of orofacial symptoms and OHRQOL in the background population. At this point, no validated examination methods exist to differentiate between “general temporomandibular disorders” and JIA-induced orofacial conditions and that constitute a limitation to the present study.

In addition, the degree of fluctuation of orofacial symptoms during the 25-month observation period is unknown, since we only examined two time-points (T0 and T1). Therefore, the term “persistent symptoms” in patients with reports of orofacial symptoms at both time points is somewhat vague and does not accurately characterize the degree of fluctuation or persistence of symptoms between these time points.

## Conclusion

Self-reported orofacial pain and functional disability were common findings in a cohort of JIA patients followed over 2 years. These symptoms seem to persist over time in most patients, and significantly reduce OHRQOL. Based on the findings of this study, we strongly recommend incorporating a standardized orofacial examination into the assessment of children diagnosed with JIA. A sudden reduction in TMJ function and/or orofacial pain should prompt increase attention and appropriate referral of the patient for further examination, and if necessary, initiation of treatment.

## Additional file

Additional file 1: Prevalence of items impacting the OHRQOL among patients with and without TMJ-arthritis symptoms. (DOCX 26 kb)

## Abbreviations

JIA: Juvenile idiopathic arthritis
OHRQOL: Oral health-related quality of life
TMJ: Temporomandibular joint
