# Supplementary material for: Orofacial symptoms and oral health-related quality of life in juvenile idiopathic arthritis: a two-year prospective observational study
Source: Pediatr Rheumatol Online J. 2018 Jul 13;16:47. doi: 10.1186/s12969-018-0259-4 (PMC6043998; doi:10.1186/s12969-018-0259-4)
Supplement: Supplementary file 1 — Prevalence of items impacting the OHRQOL among patients with and without TMJ-arthritis symptoms. (DOCX 26 kb) [file 12969_2018_259_MOESM1_ESM.docx]

| **Rahimi H et al.** | **Orofacial Symptoms and Oral Health-Related Quality of Life in Juvenile idiopathic arthritis: A two-year prospective observational study** |
| --- | --- |

**Supplementary material**

**Prevalence of items impacting the OHRQOL among patients with and without TMJ-arthritis symptoms**

| **During the last 3 months, how often have you… (had/been)…** | | | **Never** | | |  | | | **1 or 2 times** | | | | |  | | **Sometimes** | | | |  | | | | **Often** | | | | |  | | | **Every day or almost every day** | | | | **Significant** |  |  |
| --- | --- | --- | --- | --- | --- | --- | --- | --- | --- | --- | --- | --- | --- | --- | --- | --- | --- | --- | --- | --- | --- | --- | --- | --- | --- | --- | --- | --- | --- | --- | --- | --- | --- | --- | --- | --- | --- | --- |
|  | **Symp.** | | | **No symp.** | | | **Symp.** | | | | **No symp.** | | | | **Symp.** | | | **No symp.** | | | | | **Symp.** | | | | | **No symp.** | | | **Symp.** | | | **No symp.** | |  |  |  |
| **Emotional well-being** |  | | |  | | |  | | | |  | | | |  | | |  | | | | |  | | | | |  | | |  | | |  | |  |  |  |
| Irritable/frustrated | 27.4% (17/62) | | | 92.2% (47/51) | | | 35.5% (22/62) | | | | 2.0% (1/51) | | | | 19.4 % (12/62) | | | 5.9 % (3/51) | | | | | 14.5% (9/62) | | | | | 0%  (0/51) | | | 3.2% (2/62) | | | 0% (0/51) | | <0.05 |  |  |
| Insecure | 51.6 % (32/62) | | | 90.2% (46/51) | | | 24.2% (15/62) | | | | 7.8% (4/51) | | | | 14.5% (9/62) | | | 2.0% (1/51) | | | | | 8.1% (5/62) | | | | | 0%  (0/51) | | | 1.6% (1/62) | | | 0% (0/51) | | <0.05 |  |  |
| Shy/embarrassed | 62.9% (39/62) | | | 90.2% (46/51) | | | 17.7% (11/62) | | | | 7.8% (4/51) | | | | 9.7% (6/62) | | | 2.0% (1/51) | | | | | 8.1% (5/62) | | | | | 0%  (0/51) | | | 1.6% (1/62) | | | 0% (0/51) | | <0.05 |  |  |
| Concerned about what other people think***** | 61.3% (38/62) | | | 86.3% (44/51) | | | 16.1% (10/62) | | | | 5.9% (3/51) | | | | 11.3% (7/62 | | | 2.0% (1/51) | | | | | 8.1% (5/62) | | | | | 3.9% (2/51) | | | 3.2% (2/62) | | | 0% (0/51) | | <0.05 |  |  |
| Worried that is less attractive than others | 62.9% (39/62) | | | 88.2% (45/51) | | | 21.0% (13/62) | | | | 7.8% (4/51) | | | | 8.1% (5/62) | | | 2.0% (1/51) | | | | | 4.8% (3/62) | | | | | 0%  (0/51) | | | 3.2% (2/62) | | | 2.0% (1/51) | | <0.05 |  |  |
| Upset | 54.8% (34/62) | | | 90.2% (46/51) | | | 21.0% (13/62) | | | | 3.9% (2/51) | | | | 14.5% (9/62) | | | 5.9% (3/51) | | | | | 8.1% (5/62) | | | | | 0%  (0/51) | | | 1.6% (1/62) | | | 0% (0/51) | | <0.05 |  |  |
| Nervous/afraid | 80.6% (50/62) | | | 94.1% (48/51) | | | 9.7%  (6/62) | | | | 3.9% (2/51) | | | | 4.8% (3/62) | | | 2.0% (1/51) | | | | | 3.2% (2/62) | | | | | 0%  (0/51) | | | 1.6% (1/62) | | | 0% (0/51) | | <0.05 |  |  |
| Worried that is less healthy | 66.1% (41/62) | | | 94.1% (48/51) | | | 14.5% (9/62) | | | | 2.0% (1/51) | | | | 9.7% (6/62) | | | 2.0% (1/51) | | | | | 6.5% (4/62) | | | | | 2.0% (1/51) | | | 3.2% (2/62) | | | 0% (0/51) | | <0.05 |  |  |
| Worried that is different from other people | 75.8% (47/62) | | | 92.2% (47/51) | | | 14.5% (9/62) | | | | 3.9% (2/51) | | | | 3.2% (2/62) | | | 3.9% (2/51) | | | | | 1.6% (1/62) | | | | | 0%  (0/51) | | | 4.8% (3/62) | | | 0% (0/51) | | <0.05 |  |  |
| Jealous of family members | 83.9% (52/62) | | | 92.2% (47/51) | | | 8.1%  (5/62) | | | | 5.9% (3/51) | | | | 3.2% (2/62) | | | 0%  (0/51) | | | | | 3.2% (2.62) | | | | | 2.0% (1/51) | | | 1.6% (1/62) | | | 0% (0/51) | | N.S |  |  |
| Worried about having fewer friends | 93.5% (58/62) | | | 100% (51/51) | | | 4.8%  (3/62) | | | | 0%  (0/51) | | | | 0%  (0/62) | | | 0%  (0/51) | | | | | 0%  (0/62) | | | | | 0%  (0/51) | | | 1.6% (1/62) | | | 0% (0/51) | | N.S |  |  |
| **APPENDIX 1** *(continued)* | | | | | | | | |  |  | |  | | |  | |  | |  | | |  | | | |  |  | | | | | | | |  |  |  |  |
| **During the last 3 months, how often have you… (had/been)…** | | **Never** | | |  | | | **1 or 2 times** | | | | |  | | | **Sometimes** | | | | |  | | | | **Often** | | | | |  | | | **Every day or almost every day** | | | **Significant** |  | |
|  | **Symp.** | | | **No symp.** | | | **Symp.** | | | | **No symp.** | | | | **Symp.** | | | **No symp.** | | | | | **Symp.** | | | | | **No symp.** | | | **Symp.** | | | **No symp.** | |  |  |  |
| **Social well-being** |  | | |  | | |  | | | |  | | | |  | | |  | | | | |  | | | | |  | | |  | | |  | |  |  |  |
| Missed school***** | 45.2% (28/62) | | | 60.8% (31/51) | | | 29.0% (18/62) | | | | 33.3% (17/51) | | | | 16.1% (10/62) | | | 3.9% (2/51) | | | | | 8.1% (5/62) | | | | | 0%  (0/51) | | | 1.6% (1/62) | | | 0% (0/51) | | <0.05 |  |  |
| Had a hard time paying attention in school | 67.7% (42/62) | | | 100% (51/51) | | | 14.5% (9/62) | | | | 0%  (0/51) | | | | 14.5% (9/62) | | | 0%  (0/51) | | | | | 0%  (0/62) | | | | | 0%  (0/51) | | | 3.2% (2/62) | | | 0% (0/51) | | <0.05 |  |  |
| Difficulty doing homework | 71.0% (44/62) | | | 98.0 % (50/51) | | | 21.0% (13/62) | | | | 2.0% (1/51) | | | | 4.8% (3/62) | | | 0%  (0/51) | | | | | 1.6% (1/62) | | | | | 0%  (0/51) | | | 1.6% (1/62) | | | 0% (0/51) | | <0.05 |  |  |
| Not wanted to speak/read out loud in class | 87.1% (54/62) | | | 100% (51/51) | | | 8.1%  (5/62) | | | | 0%  (0/51) | | | | 1.6% (1/62) | | | 0%  (0/51) | | | | | 3.2% (2/62) | | | | | 0%  (0/51) | | | 0%  (0/62) | | | 0% (0/51) | | <0.05 |  |  |
| Not wanted/unable to take part in activities (clubs, drama, music)***** | 92.0% (57/62) | | | 100% (51/51) | | | 1.6%  (1/62) | | | | 0%  (0/51) | | | | 4.8% (3/62) | | | 0%  (0/51) | | | | | 0%  (0/62) | | | | | 0%  (0/51) | | | 0%  (0/62) | | | 0% (0/51) | | N.S |  |  |
| Not wanted to talk to other children | 88.7% (55/62) | | | 100% (51/51) | | | 8.1%  (5/62) | | | | 0%  (0/51) | | | | 0%  (0/62) | | | 0%  (0/51) | | | | | 3.2% (2/62) | | | | | 0%  (0/51) | | | 0%  (0/62) | | | 0% (0/51) | | <0.05 |  |  |
| Avoided smiling when around other children | 79.0% (49/62) | | | 96.1% (49/51) | | | 8.1%  (5/62) | | | | 3.9% (2/51) | | | | 8.1% (5/62) | | | 0%  (0/51) | | | | | 3.2% (2/62) | | | | | 0%  (0/51) | | | 1.6% (1/62) | | | 0% (0/51) | | <0.05 |  |  |
| Difficulty playing musical instrument***** | 92.0% (57/62) | | | 100% (51/51) | | | 3.2%  (2/62) | | | | 0%  (0/51) | | | | 1.6% (1/62) | | | 0%  (0/51) | | | | | 0%  (0/62) | | | | | 0%  (0/51) | | | 0%  (0/62) | | | 0% (0/51) | | <0.05 |  |  |
| Not wanted/unable to spend time with other children***** | 93.5% (58/62) | | | 100% (51/51) | | | 3.2%  (2/62) | | | | 0%  (0/51) | | | | 1.6% (1/62) | | | 0%  (0/51) | | | | | 0%  (0/62) | | | | | 0%  (0/51) | | | 0%  (0/62) | | | 0% (0/51) | | N.S |  |  |
| Argued with family members or other children | 90.3% (56/62) | | | 98.0 % (50/51) | | | 6.5%  (4/62) | | | | 2.0% (1/51) | | | | 3.2% (2/62) | | | 0%  (0/51) | | | | | 0%  (0/62) | | | | | 0%  (0/51) | | | 0%  (0/62) | | | 0% (0/51) | | N.S |  |  |
| Teased/called names by other children | 98.4/ (61/62) | | | 100% (51/51) | | | 1.6%  (1/62) | | | | 0%  (0/51) | | | | 0%  (0/62) | | | 0%  (0/51) | | | | | 0%  (0/62) | | | | | 0%  (0/51) | | | 0%  (0/62) | | | 0% (0/51) | | N.S |  |  |
| Left out by other children | 96.8% (60/62) | | | 100% (51/51) | | | 3.2%  (2/62) | | | | 0%  (0/51) | | | | 0%  (0/62) | | | 0%  (0/51) | | | | | 0%  (0/62) | | | | | 0%  (0/51) | | | 0%  (0/62) | | | 0% (0/51) | | N.S |  |  |
| Asked questions by other children about the condition ***** | 50% (31/62) | | | 82.4% (42/51) | | | 25.8% (16/62) | | | | 9.8% (5/51) | | | | 17.7% (11/62) | | | 5.9% (3/51) | | | | | 6.5% (4/62) | | | | | 0%  (0/51) | | | 0%  (0/62) | | | 0% (0/51) | | <0.05 |  |  |
| Avoided eating with other children | 92.0% (57/62) | | | 100% (51/51) | | | 3.2%  (2/62) | | | | 0%  (0/51) | | | | 3.2% (2/62) | | | 0%  (0/51) | | | | | 1.6% (1/62) | | | | | 0%  (0/51) | | | 0%  (0/62) | | | 0% (0/51) | | <0.05 |  |  |
| Not wanted/unable to take part in family activities | 95.2% (59/62) | | | 100% (51/51) | | | 3.2%  (2/62) | | | | 0%  (0/51) | | | | 1.6% (1/62) | | | 0%  (0/51) | | | | | 0%  (0/62) | | | | | 0%  (0/51) | | | 0%  (0/62) | | | 0% (0/51) | | N.S |  |  |
| Not allowed by parents to join other children | 93.5% (58/62) | | | 98.0 % (50/51) | | | 4.8% (3/62) | | | | 0% (0/51) | | | | 0% (0/62) | | | 0% (0/51) | | | | | 0% (0/62) | | | | | 0% (0/51) | | | 0% (0/62) | | | 2.0% (1/51) | | N.S |  |  |
| Not wanted/unable to go out with family | 100% (62/62) | | | 100% (51/51) | | | 0%  (0/62) | | | | 0%  (0/51) | | | | 0%  (0/62) | | | 0%  (0/51) | | | | | 0%  (0/62) | | | | | 0%  (0/51) | | | 0%  (0/62) | | | 0% (0/51) | | N.S |  |  |
| Not wanted/unable to go to family gatherings***** | 98.4/ (61/62) | | | 98.0 % (50/51) | | | 0%  (0/62) | | | | 0%  (0/51) | | | | 1.6% (1/62) | | | 0%  (0/51) | | | | | 0%  (0/62) | | | | | 0%  (0/51) | | | 0%  (0/62) | | | 0% (0/51) | | N.S |  |  |

***** One or two missing values
